# Role of Chaperone-Mediated Autophagy Dysfunctions in the Pathogenesis of Parkinson’s Disease

**DOI:** 10.3389/fnmol.2016.00157

**Published:** 2016-12-23

**Authors:** Gessica Sala, Daniele Marinig, Alessandro Arosio, Carlo Ferrarese

**Affiliations:** ^1^Laboratory of Neurobiology, School of Medicine and Surgery, Milan Center for Neuroscience (NeuroMI), University of Milano-BicoccaMonza, Italy; ^2^PhD Program in Neuroscience, University of Milano-BicoccaMonza, Italy; ^3^Department of Neurology, San Gerardo Hospital, University of Milano-BicoccaMonza, Italy

**Keywords:** chaperone-mediated autophagy, Parkinson’s disease, alpha-synuclein, lamp2A, hsc70, MEF2D

## Abstract

Chaperone-mediated autophagy (CMA) represents a selective form of autophagy involved in the degradation of specific soluble proteins containing a pentapeptide motif that is recognized by a cytosolic chaperone able to deliver proteins to the lysosomes for degradation. Physiologically, CMA contributes to maintain crucial cellular functions including energetic balance and protein quality control. Dysfunctions in CMA have been associated to the pathogenesis of several neurodegenerative diseases characterized by accumulation and aggregation of proteins identified as CMA substrates. In particular, increasing evidence highlights the existence of a strong relationship between CMA defects and Parkinson’s disease (PD). Several mutations associated with familial forms of PD (SNCA, LRRK2, UCHL1 and DJ-1) have been demonstrated to block or reduce the activity of CMA, the main catabolic pathway for alpha-synuclein (asyn). CMA dysfunctions also leads to a mislocalization and inactivation of the transcription factor MEF2D that plays a key-role in the survival of dopaminergic neurons. Furthermore, reduced levels of CMA markers have been observed in post mortem brain samples from PD patients. The aim of this review article is to provide an organic revision of evidence for the involvement of CMA dysfunctions in the pathogenesis of PD. Updated findings obtained in patient’s specimens will be resumed, and results deriving from *in vivo* and *in vitro* studies will be discussed to evidence the current knowledge on the molecular mechanisms underlying CMA alterations in PD. Finally, the possibility of up-regulating CMA pathway as promising neuroprotective strategy will be considered.

## Parkinson’s Disease Is a Proteinopathy

Parkinson’s disease (PD), as well as other neurodegenerative diseases, belongs to the large category of proteinopathies, conditions characterized by the presence of proteinaceous inclusions within the degenerating neurons. The identification of such aggregates supports the view that misfolded proteins represent a basic requirement for the neurodegenerative process and provided input to verify the existence of possible dysfunctions of the biological systems influencing neuronal protein homeostasis. Considering the post-mitotic nature of neurons, a proper activity of the intracellular protein degradation systems appears to be crucial to ensure the maintenance of cell homeostasis and to prevent the onset of the neurodegeneration.

In PD, alpha-synuclein (asyn) has been proposed as the central pathogenic protein, based on its identification as the main component of the intraneuronal aggregates, known as Lewy bodies, that represent a neuropathological hallmark of PD (Spillantini et al., 1997). The accumulation of aggregated asyn has been related to defects in the two major protein catabolic systems (Xilouri et al., 2013b), the ubiquitin-proteasome system (UPS) and the autophagy-lysosome pathway (ALP), represented by macroautophagy, chaperone-mediated autophagy (CMA) and microautophagy.

Currently, the exact contribution of UPS and ALP to asyn degradation remains unclear. There is a general consensus on the view that, while UPS degrades only small and soluble oligomers of asyn, ALP becomes crucial in pathologic conditions (Ebrahimi-Fakhari et al., 2011). Furthermore, as the activity of UPS and ALP appears functionally connected, dysfunctions in one of these systems directly influence the other (Ding et al., 2007; Pandey et al., 2007; Korolchuk et al., 2009; Qiao and Zhang, 2009). Alterations of both UPS and macroautophagy have been reported in PD patients. Specifically, structural and functional alterations in the 20S proteasome subunit was demonstrated in post mortem substantia nigra of patients with sporadic PD (McNaught and Olanow, 2003), and an increased number of autophagic vacuoles was found in melanized neurons of the substantia nigra in PD patients (Anglade et al., 1997). After the demonstration that the efficiency of CMA pathway is crucial in regulating the intraneuronal levels of asyn (Cuervo et al., 2004; Mak et al., 2010), dysfunctions in this selective catabolic pathway have been identified as important pathogenic contributors to PD, as discussed in the following sections.

## Molecular Characteristics of CMA and Physiological Functions

**Figure 1:**
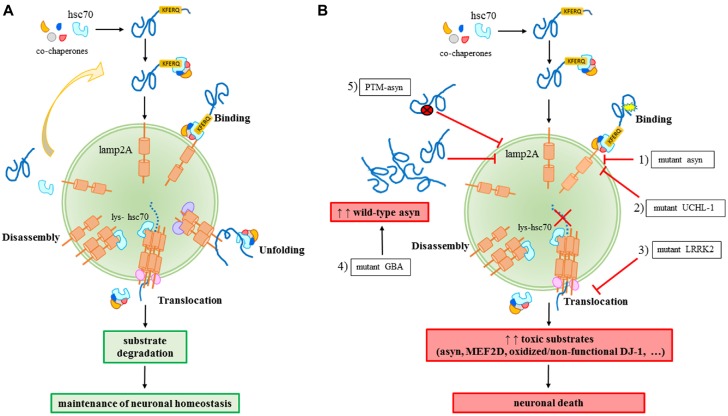
**Chaperone-mediated autophagy (CMA) in physiology (A) and in Parkinson’s disease (PD)-related conditions** (B)**. (A)** A proper CMA activity contributes to the maintenance of the neuronal homeostasis; **(B)** different alterations related to familial or sporadic PD forms impair CMA and significantly contribute to neuronal death: (1) mutant A30P and A53T asyn bind to lamp2A with a higher affinity, thus inhibiting the binding of other substrates; (2) mutant I93M UCHL-1 abnormally interacts with lamp2A causing asyn accumulation; (3) mutant LRRK2 prevents lamp2A multimerization and consequently, the translocation of substrates; (4) mutant GBA may increase the levels of wild-type asyn resulting in a block of CMA due to an excess of substrates; (5) PTM (post-translational-modified)-asyn forms, including dopamine-modified, oxidized, phosphorylated and nitrated asyn, inhibit CMA-mediated degradation of asyn and other substrates.

CMA is a selective catabolic pathway which allows the degradation of specific cellular proteins. This feature makes CMA an efficient way to remove specific damaged or abnormal proteins and becomes an essential key-regulator in multiple cellular processes. Substrates eligible to be degraded by this pathway, including asyn, contains in their sequence a pentapeptide conserved motif biochemically related to KFERQ (Dice, 1990). This motif is selectively recognized by the cytosolic chaperone heat shock cognate protein of 70 kDa, hsc70, which binds the substrate and translocates it to the lysosome membrane (Chiang et al., 1989). Hsc70, in association with a specific subset of molecular co-chaperones, participates to the unfolding of the substrate protein, an essential requirement for translocation to the lysosomes (Agarraberes and Dice, 2001). After the binding to the chaperones, the substrate is targeted to the lysosomal membrane where it interacts with the lysosome-associated membrane protein type 2A (lamp2A; Cuervo and Dice, 1996). Lamp2A, in association with other proteins, forms a multi-protein complex and regulates the substrate translocation into the lumen of the lysosome where substrate will be degraded (Bandyopadhyay et al., 2008). The translocation of the substrate across the lysosomal membrane requires the presence of a specific form of hsc70 resident in the lysosomes (lys-hsc70) which, at the end of the process, actively disassembles lamp2A into monomers to initiate a new cycle of substrate uptake and degradation (Agarraberes and Dice, 2001; Bandyopadhyay et al., 2008). Consequently, the rate of CMA can be modulated by the rate of assembly/disassembly of the translocation complex and, for this reason, lamp2A could be considered the rate-limiting protein of the process (Figure 1A).

Although CMA can assume some tissue-specific functions (discussed below), this pathway carries out two main general functions in all cell types and, indeed, basal levels of CMA activity can be detected in almost all cells. The first role proposed for CMA is an involvement in amino acids recycling during starvation, a stress condition characterized by energy depletion. In this circumstance, CMA is strongly activated to break down non-essential substrates to sustain synthesis of vital proteins in the absence of nutrients (Cuervo et al., 1995a). The other important cellular function of CMA is protein quality control based on its ability to selectively remove specific proteins from the cytosol. Indeed, different subunits of the catalytic core of the proteasome have been shown to be degraded in lysosomes by this pathway (Cuervo et al., 1995b). Moreover, CMA is the principal catabolic process by which oxidized, aberrant and damaged proteins, as well as proteins prone to aggregate, are removed from the cell. For this reason, CMA is strongly up-regulated during oxidative stress (Kiffin et al., 2004) or exposure to toxic compounds (Cuervo et al., 1995a, 1999), and cells with reduced CMA activity are more susceptible to oxidative agents (Massey et al., 2006).

In addition to these general functions, CMA regulates other cell type-specific functions such as antigen presentation in immune cells (Zhou et al., 2005), control of renal tubular cells growth through the degradation of the Pax2 transcription factor (Sooparb et al., 2004), and preservation of neuronal viability (Yang et al., 2009). In particular, the selective degradation of a neuronal survival transcriptional factor, myocyte enhancer factor 2D (MEF2D) has been described to be ascribable, at least in part, to CMA and this represents a fundamental mechanism to ensure an adequate neuronal response to injury (Yang et al., 2009). Indeed, many evidences directly sustain a MEF2D involvement in PD pathogenesis: reduced MEF2D levels were observed in brains of patients, in PD animal models and in response to asyn accumulation and aggregation (Yang et al., 2009; Chu et al., 2011), although unchanged MEF2D expression was observed in lymphomonocytes obtained from PD patients (Sala et al., 2014). Moreover, She et al. (2011) identified a mitochondrial form of MEF2D with decreased levels in PD brains and in animal models that were treated with PD-related toxins. The exact mechanisms for MEF2D alterations that leads to neurodegeneration are still largely unknown, but a possible contribution of oxidative stress has been hypothesized. Indeed, 6-hydroxydopamine (6-OHDA) is able to directly oxidize MEF2D and increased oxidized MEF2D levels were observed in PD brains (Gao et al., 2014). Moreover, oxidized MEF2D binds hsc70 with higher affinity (Gao et al., 2014), and oxidative stress induces lamp2A up-regulation that in turn increases MEF2D degradation (Kiffin et al., 2004). The role of MEF2D down-regulation in the pathogenesis of PD is also confirmed by the evidence that PD-related toxins reduce the expression of this neuronal transcription factor (Wang et al., 2014), and a potential pharmacological strategy using activators of MEF2D ameliorates the pathological outcome in PD animal models (Yao et al., 2012). The possible beneficial role of MEF2D induction was also confirmed in an *in vitro* model after an acute treatment with low doses of the mitochondrial complex I inhibitor rotenone: a *de novo* synthesis of MEF2D was observed to increase the nuclear fraction of this pro-survival factor to compensate the mild cellular damage (Sala et al., 2013).

## Alterations of CMA in PD

### Effect of PD-Related Genetic Mutations on CMA

For years, PD was considered a pure sporadic disease caused by environmental factors such as chemicals and head injuries. However, it was increasingly clear that environmental factors were not enough to cause the disease. SNCA, which encodes for asyn, was the first gene discovered to be associated to autosomal dominant forms of PD. Actually, single nucleotide variants, duplications and triplications on the asyn gene cause an early onset of the disease (Polymeropoulos et al., 1997; Singleton et al., 2003; Chartier-Harlin et al., 2004). In particular, the overexpression rate was correlated to the disease’s severity, with patients carrying a triplication of SNCA gene developing a more severe form of the disease (Singleton et al., 2003) than the duplication cases. Later on, other mutations were identified to cause familial forms of PD. Among them, transgenic cellular and animal models demonstrated an association of such mutations to dysfunctions of CMA. Mutant A30P and A53T asyn bind to lamp2A with a higher affinity without being internalized and degraded, thus preventing the binding of other substrates (Cuervo et al., 2004). Pathogenic mutations of the leucine-rich repeat kinase 2 (LRRK2), including the most common G2019S mutant, can block CMA by inhibiting the translocation complex at the lysosome membrane (Orenstein et al., 2013). The net result of both these aberrant interactions is the accumulation of toxic substrates leading to a generalized cellular stress. Abnormal interaction with CMA components, including lamp2A and the chaperones hsc70 and heat shock protein 90 (hsp90), has been also demonstrated for the PD-associated I93M mutant ubiquitin C-terminal hydrolase L1 (UCH-L1), which results in an increase of asyn levels within cells due to a blockage of its degradation via CMA (Kabuta et al., 2008). Recently, PARK7/DJ-1, a protein implied in antioxidative defense and mitochondrial homeostasis maintenance and whose mutations are responsible for autosomal dominant forms of PD, has been identified as a substrate of CMA (Wang et al., 2016). It has also been found that inactive DJ-1 forms increase following CMA inhibition, and this contributes to mitochondrial dysfunctions. In particular, it has been demonstrated that oxidized/non-functional DJ-1 forms are preferentially degraded by this pathway. This consequently can regulate mitochondrial function via maintaining DJ-1 homeostasis (Wang et al., 2016). Finally, emerging data suggest a role for mutations of GBA gene in autophagy alterations. GBA mutations cause Gaucher’s disease leading to a deficiency of the lysosomal enzyme glucocerebrosidase (GCase) and they are recognized as important risk factors for PD (Sidransky and Lopez, 2012; Migdalska-Richards and Schapira, 2016). As a matter of fact, the functional loss of GCase in primary cultures or human iPS neurons causes accumulation of asyn which may result in a CMA impairment (Mazzulli et al., 2011; Figure 1B).

### Evidence for CMA Dysfunction in Sporadic PD

The involvement of CMA in PD pathogenesis is not only supported by genetic evidences but also alterations of CMA parameters were also observed in sporadic forms of PD. Post mortem brain samples from PD patients revealed a down-regulation of both hsc70 and lamp2A in the substantia nigra pars compacta and amygdala as compared to healthy controls (Alvarez-Erviti et al., 2010). Furthermore, the selective loss of lamp2A protein in early stages of PD correlated with increased levels of asyn and other cytosolic CMA substrate proteins (Murphy et al., 2015), suggesting that CMA dysfunctions may precede asyn pathogenic accumulation in PD. Interestingly, hsc70 down-regulation was also demonstrated in peripheral lymphomonocytes of sporadic PD patients (Sala et al., 2014; Papagiannakis et al., 2015). In these cells, a reduction of lamp2 (not lamp2A) protein levels and an increase of the autophagosome marker LC3-II were also reported (Prigione et al., 2010; Wu et al., 2011). This suggests the existence of a systemic alteration of autophagy in PD. Moreover, the sequencing of genomic DNAs from leukocytes of sporadic PD patients allowed the identification of a novel sequence variant in a patient that significantly reduced the transcriptional activities of lamp2 gene promoter (Pang et al., 2012). Since lamp2A is the splicing isoform of lamp2 specific for CMA, reduced levels of lamp2 may be reasonably associated to an abatement of CMA activity. Recently, the potential role of microRNAs (miRNAs) in the CMA down-regulation observed in PD patients was also found. Indeed, the down-regulation of hsc70 and lamp2A in PD brains has been shown to correlate with an up-regulation of six miRNAs targeting the 3′ UTR of hsc70 or lamp2A (Alvarez-Erviti et al., 2013). However, currently there is a dearth of study material regarding the role of “CMA miRNAs” in PD pathogenesis apart from the only one study that showed the up-regulation of one of them resulted in the knock-down of hsc70 and increased levels of asyn in human SH-SY5Y neuroblastoma cells (Li et al., 2014).

### Alterations of CMA in Cellular and Animal Models of PD

Collectively, CMA alterations are considered as an important pathogenic mechanism responsible in nigral neuronal death, even though the underlying molecular mechanisms are not yet fully explained. CMA impairment may favor the processes that are known to play a major role in PD pathology, such as asyn toxicity, mitochondrial dysfunction and oxidative stress, which in turn have been demonstrated to negatively influence autophagy activity, thus originating a vicious circle whose final outcome is the death of dopaminergic neurons (Bandopadhyay and de Belleroche, 2010).

The expression of A53T mutant asyn is able to cause a CMA impairment, as observed in adenoviral transfected PC12 and SH-SY5Y cells (Xilouri et al., 2009). Such impairment leads to a global lysosomal dysfunction that may be counteracted by a compensatory up-regulation of macroautophagy. Experimental models of selective CMA blockage also support the potential fundamental role of CMA in PD pathogenesis. The down-regulation of lamp2A receptor resulted in an increased sensitivity to oxidative stressors in mouse fibroblasts (Massey et al., 2006) and in the accumulation of soluble high molecular weight and detergent-insoluble species of asyn in PC12 and SH-SY5Y cells (Vogiatzi et al., 2008). Lamp2A down-regulation also causes a raise in autophagic vacuoles within rats nigral neurons with a progressive loss of the same neurons as well as unilateral motor behavioral deficits (Xilouri et al., 2016). However, other studies failed to report CMA dysfunctions after lamp2 silencing, and a compensatory activation of other proteolytic pathways was reported after the aspecific down-regulation of all lamp2 isoforms (Eskelinen et al., 2004; Rothaug et al., 2015). Recently, it has been observed that RNA interference against hsc70 results in an up-regulation of asyn mRNA and protein levels, a condition that is likely to favor protein accumulation and aggregation typical of PD (Sala et al., 2016), suggesting again that the observed down-regulation of CMA effectors observed in PD brains may be a key-condition for a deadly accumulation of toxic substrates, especially asyn, within dopaminergic neurons. Post-translational modifications of wild-type asyn, such as oxidation, phosphorylation and nitration, are known to alter CMA activity, thus resulting in an inhibition of CMA-mediated asyn degradation, while dopamine-modification of asyn also impairs the degradation of other CMA substrates (Martinez-Vicente et al., 2008; Figure 1B). These modifications were largely found in PD brains (Fujiwara et al., 2002; Anderson et al., 2006) as well as in peripheral samples from patients (Prigione et al., 2010; Foulds et al., 2011).

Furthermore, PD-related toxins, such as rotenone and paraquat, are able to induce alterations of CMA effectors and substrates. Actually, midbrains of paraquat-treated mice showed a CMA activation, as indicated by increased levels of lamp2A and hsc70, and an enhanced lysosomal clearance of asyn (Mak et al., 2010). On the contrary, exposure to the pesticide rotenone caused an up-regulation of asyn in SH-SY5Y cells (Sala et al., 2013) and a down-regulation of hsc70 in SH-SY5Y cells and mouse cortical neurons (Sala et al., 2016). These different results may be explained by the mechanisms of action of rotenone, a mitochondrial complex I inhibitor, and paraquat, an oxidative stress donor. As a matter of fact, CMA seems to be up-regulated in oxidative stress conditions, in order to participate in the removal of oxidized proteins. Rats exposed to sub-lethal doses of paraquat or 6-OHDA showed a significant increase in the expression of lamp2A and hsp90 (Kiffin et al., 2004; Marin and Aguilar, 2011) that can be interpreted as a cellular conservative mechanism for the removal of oxidized proteins. Impaired macroautophagy in knocked-out Atg5 mouse embryonic fibroblasts resulted in an up-regulation of CMA (Kaushik et al., 2008) that exerts a protective role against toxicity induced by menadione and UV light but not by staurosporine and endoplasmic reticulum stress (Wang et al., 2008). Similarly, up-regulation of CMA in Atg5 null murine embryonic fibroblasts increased cellular resistance to photokilling, while CMA-deficient cells were protected against the endoplasmic reticulum stressor thapsigargin (Dewaele et al., 2011). Collectively, these findings unveil a cytoprotective role of CMA against specific stressors with a dominant role of scavenger of oxidized proteins, a pivotal self-preserving function for the nigral dopaminergic neurons.

## Up-Regulation of CMA as Potential Therapeutic Strategy for PD

Given the evidences of the critical role of CMA activity in controlling the levels of potentially toxic substrates, the up-regulation of such autophagic pathway represents a new challenge for the development of therapeutic strategies for PD and other neurodegenerative disorders characterized by protein accumulation, such as Alzheimer’s disease, amyotrophic lateral sclerosis and Huntington’s disease. Early attempts with chemical modulators identified small molecules that are able to induce CMA such as 6-aminonicotinamide and geldanamycin, inhibitors of the glucose-6-phopshate dehydrogenase or hsp90, respectively (Finn et al., 2005). More recently, it has been demonstrated that signaling through retinoic acid receptor alpha (RARalpha) inhibits CMA and synthetic derivatives of all-trans-retinoic acid reverse this inhibitory effect protecting cells against oxidation and proteotoxicity (Anguiano et al., 2013). Another promising strategy for CMA induction involves the direct overexpression of lamp2A. In a first attempt on transgenic mice, up-regulating the lysosomal receptor of CMA improved liver function and slowed-down cellular damage associated with proteotoxicity (Zhang and Cuervo, 2008). This paradigm has been recently applied to *in vitro* and *in vivo* neuronal systems. Lamp2A overexpression in human neuroblastoma SH-SY5Y cells and rat primary cortical neurons protected cells from asyn-induced neurotoxicity (Xilouri et al., 2013a). Similarly, this strategy restored the asyn-mediated nigrostriatal degeneration and reduced total asyn levels as well as the generation of aggregated and phosphorylated asyn forms in rats (Xilouri et al., 2013a). The up-regulation of lamp2A may also be obtained through the modulation of miRNAs. For example, miR-21 overexpression down-regulates the expression of lamp2A (Alvarez-Erviti et al., 2013), while its inhibition up-regulates lamp2A levels and reduces asyn levels in SH-SY5Y cells and MPTP-induced PD mice (Su et al., 2016). Finally, given its role as CMA carrier and its anti-aggregant properties on asyn (Pemberton et al., 2011; Pemberton and Melki, 2012), hsc70 may represent a further potential target for PD therapeutic strategies, but no studies have yet explored such possibility.

## Author Contributions

GS: conception of the whole content and organization of the mini-review, writing of the introductive section, assembly of the different sections, figure arrangement and overall revision; DM: writing of the sections on PD-related alterations of CMA and CMA-related therapeutic strategies; AA: writing of the section on physiology of CMA and figure preparation; CF: overall revision.

## Conflict of Interest Statement

The authors declare that the research was conducted in the absence of any commercial or financial relationships that could be construed as a potential conflict of interest.
